# Deciphering the Hearts: Geometric Morphometrics Reveals Shape Variation in *Abatus* Sea Urchins across Subantarctic and Antarctic Seas

**DOI:** 10.3390/ani14162376

**Published:** 2024-08-16

**Authors:** Fernando Moya, Jordan Hernández, Manuel J. Suazo, Thomas Saucède, Paul Brickle, Elie Poulin, Hugo A. Benítez

**Affiliations:** 1 Millennium Institute Biodiversity of Antarctic and Subantarctic Ecosystems (BASE), Santiago 7800003, Chileepoulin@uchile.cl (E.P.);; 2 Laboratorio de Ecología Molecular, Departamento de Ciencias Ecológicas, Facultad de Ciencias, Universidad de Chile, Santiago 7800003, Chile; 3 Laboratorio de Ecología y Morfometría Evolutiva, Centro de Investigación de Estudios Avanzados del Maule, Universidad Católica del Maule, Talca 3466706, Chile; 4 Programa de Doctorado en Salud Ecosistémica, Centro de Investigación de Estudios Avanzados del Maule, Universidad Católica del Maule, Talca 3466706, Chile; 5 Cape Horn International Center (CHIC), Centro Universitario Cabo de Hornos, Universidad de Magallanes, Puerto Williams 6350000, Chile; 6 Instituto de Alta Investigación, Universidad de Tarapacá, Casilla 7D, Arica 1010069, Chile; 7 Biogeosciences UMR 6282 CNRS, Université de Bourgogne, EPHE, 21078 Dijon, France; 8 South Atlantic Environmental Research Institute, Falkland Islands, Port Stanley FIQQ 1ZZ, UK

**Keywords:** echinoderm, invertebrates, geometric morphometrics, shape, Spatangoida, Southern Ocean

## Abstract

**Simple Summary:**

*Abatus* is a genus of sea urchin that inhabits the Southern Ocean. Among the 11 described species, three shared morphological traits and live in intertidal zones in Patagonia (*A. cavernosus*), Kerguelen (*A. cordatus*), Antarctica, and Tierra del Fuego (*A. agassizii*). The relationships between *Abatus* species are complicated and have not been clarified yet. This study analyzed shape variation among these species. The shape of 72 individuals from four locations in the South Shetlands, Kerguelen, Patagonia, and Falklands/Malvinas, were evaluated. Differences in shape were found in all four locations. Especially, the Falklands/Malvinas group showed a marked difference in shape compared to other localities. The possibility that the Falklands/Malvinas group shows phenotypic plasticity or represents a distinct evolutionary unit is discussed. Finally, the methodology used in this study proved to be a powerful tool to differentiate these species, highlighting its utility in systematic studies.

**Abstract:**

*Abatus* is a genus of irregular brooding sea urchins to the Southern Ocean. Among the 11 described species, three shared morphological traits and present an infaunal lifestyle in the infralittoral from the Subantarctic province; *A. cavernosus* in Patagonia, *A. cordatus* in Kerguelen, and *A. agassizii* in Tierra del Fuego and South Shetlands. The systematic of *Abatus*, based on morphological characters and incomplete phylogenies, is complex and largely unresolved. This study evaluates the shape variation among these species using geometric morphometrics analysis (GM). For this, 72 individuals from four locations; South Shetlands, Kerguelen, Patagonia, and Falklands/Malvinas were photographed, and 37 landmarks were digitized. To evaluate the shape differences among species, a principal component analysis and a Procrustes ANOVA were performed. Our results showed a marked difference between the Falklands/Malvinas and the other localities, characterized by a narrower and more elongated shape and a significant influence of location in shape but not sex. Additionally, the effect of allometry was evaluated using a permutation test and a regression between shape and size, showing significant shape changes during growth in all groups. The possibility that the Falklands/Malvinas group shows phenotypic plasticity or represents a distinct evolutionary unit is discussed. Finally, GM proved to be a powerful tool to differentiate these species, highlighting its utility in systematic studies.

## 1. Introduction

*Abatus* Troschel, 1851 is a genus of irregular sea urchin belonging to the order Spatangoida, family Schizasteridae, to which eleven extant species are currently attributed based on morphological characters; all of them are mainly distributed in the Southern Ocean [1]. Test shape in irregular sea urchins is characterized by both a bilateral and a radial symmetry that is overlaid on each other, spatangoids being commonly referred to as heart urchins given the typical outline of the test [2]. Species of *Abatus* are deposit feeders, inhabiting muddy and sandy environments at different water depths, from the shallow subtidal area down to 1000 m [1]. Species in the genus have a direct development, with no larval phase, the females brooding their young inside four aboral depressed petals (named marsupia) for several months before releasing the juveniles on the seafloor [3,4].

Four of the eleven species of *Abatus* are reported from the subantarctic province: *Abatus agassizii* Mortensen, 1910 off Tierra del Fuego, *Abatus cavernosus* Philippi, 1845 off the coasts of eastern Patagonia, *Abatus cordatus* Verrill, 1876 a species endemic to the Kerguelen Islands and plateau, and *Abatus philippii* Lovén, 1871 in the Southwestern Atlantic Ocean as far north as Mar del Plata [5]. The remaining seven species are only distributed around the Antarctic continent: *Abatus beatriceae* Larrain, 1985, *Abatus bidens* Mortensen, 1910, *Abatus curvidens* Mortensen, 1936, *Abatus elongatus* Koehler, 1908, *Abatus ingens* Koehler, 1926, *Abatus nimrodi* Koehler, 1911, and *Abatus shackletoni* Koehler, 1911 [1]. Although *A. agassizii* has an Antarctic and subantarctic distribution, it is morphologically and genetically closely related to the subantarctic species *A. cordatus* and *A. cavernosus*, suggesting that its presence in the Antarctic region is likely the result of recent colonization from northern regions [6].

These three species show biological and ecological similarities. They have the same infaunal mode of life, form dense populations with patchy distributions in medium and fine-grained sediments in the infralittoral zone at very shallow depths, and feed on detritus and organic matter by ingesting surface sediments [4,7,8]. For these reasons, they are considered keystone species in benthic marine ecosystems by highly affecting sediment and water biochemical interactions, including carbon cycling, sediment oxygenation, and inorganic nutrient fluxes, maintaining microbial and infaunal diversity [8,9]. Furthermore, they have an annual reproductive cycle, releasing their young in the summer, probably to take advantage of the increase in primary productivity, unlike the Antarctic species, which have a continuous year-long reproductive cycle [10,11].

The systematics of the genus *Abatus* have been questioned in former studies [6,12,13]. Species identification and taxonomy only rely on discrete, categorical, and linear morphological characters, e.g., [14], such as the type of pedicellariae, relative length of petals, labrum size, number of gonopores and type of fascioles [1,15]. Traditional morphometrics has been used in former studies to differentiate between species of *Abatus*: Shinner and McClintock [16] compared the size of marsupia between *A. nimrodi* and *A. shackletoni*, David et al. [12] used traditional morphometrics to differentiate between two species, namely, *A. cordatus* and *A. bidens*. Additionally, Gil et al. [4,17] evaluated size frequency and relationships between size and reproductive traits in *A. cordatus*.

Geometric morphometrics (GM) is a tool that makes it possible to study the shape variation and its covariation due to other variables, capturing the morphological structure [18]. This approach uses the analysis of the geometric coordinates of anatomical homologous points (landmarks) located on individuals or samples, allowing for the visualization of differences, evaluation of asymmetry, or identification of allometry between complex shapes [19]. Through this tool, it is possible to evaluate biological patterns, evolutionary and developmental processes, and systematic studies, analyzing subtle morphological variation between closely related species, comparing morphological adaptations to different habitats and modes of life, or shaping variation during ontogeny, both in extant and extinct fossil species [20,21,22]. Using GM in echinoderm is not very common; however, Martín-Ledo et al. [23] used shape analyses to distinguish between genera within the Cassidulidae family by analyzing the cryptic morphology of plate shapes in Echinoidea. Hernández-Díaz et al. [22], on the other hand, combine GM techniques with a molecular approach using the ophiuroid species *Ophiothrix angulate*, the differentiation of dorsal and ventral arm plates, along with integrative species delimitation analyses, identifies one of these lineages as a confirmed candidate species. De-los-Palos-Peña et al. [24] utilized a combination of scanning electron microscopy and ontogenetic studies of the odontophore in *Luidia superba* to explore patterns of size and shape variation. In addition, GM has also been used to better understand the complex interplay between genetics, phenotypes, development, and environmental constraints [25,26].

Few studies have used GM to analyze shape variation in species of *Abatus* [27]. The use of GM has proved relevant to reveal morphological differences between populations and cryptic species of echinoderms [22]. Considering the complicated systematics of the genus *Abatus* and the high morphological similarity of species, the present work aims to analyze shape differences in test outlines using GM between the three closely related species *A. agassizii*, *A. cordatus,* and *A. cavernosus*, which show similar test morphologies and biological traits.

## 2. Materials and Methods

**Figure 1:**
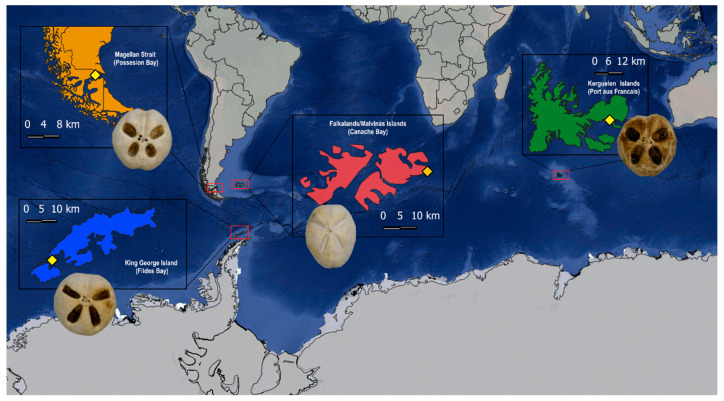
Localities where specimens of *Abatus* were sampled for this study. *Abatus cordatus* from the Kerguelen Islands (green), *Abatus cavernosus* from Patagonia (orange), *Abatus agassizii* from King George Island (blue), and *Abatus cavernosus* from the Falklands/Malvinas (red).

Seventy-two individuals belonging to three species of the genus *Abatus* were collected from four localities (Figure 1): the Kerguelen Islands (Port aux Français; 49.3510° S, 70.2107° E) (*A. cordatus*, 10 males and 10 females), the Magellan Strait (Possession Bay; 52.3353° S, 69.4782° W) in Patagonia (*A. cavernosus*, 10 males and 10 females), and King George Island (Fildes Bay; 62.2199° S, 58.9533° W) in Antarctica (*A. agassizii*, 11 males and 10 females). On the other hand, there are few studies on the genus *Abatus* in the Falkland/Malvinas Islands and discrepancies in the species that inhabit there [28]. Therefore, to include intraspecific variation, individuals from Falklands/Malvinas Islands (Canache Bay; 51.6963° S, 57.7851° W; sub-Antarctic province, 11 males), identified as *A. cavernosus*, were used in the analyses as the fourth location. Samples were collected during several field campaigns and preserved in 95% ethanol. To expose the test, individuals were submerged in a chlorine solution of 5% for one minute, and the spines were removed using a brush [27]. Species identification was performed following the most updated taxonomic key “Antarctic Echinoidea” “http://echinoidea-so.identificationkey.org/mkey.html accessed on 21 May 2024)”, where sea urchins were observed and species identified based on diagnostic morphological descriptors.

Each sample was photographed in aboral view using a Nikon d7500 digital camera. The files were converted to tps format using tpsUtil32 software v. 1.78 [29]. Following the methodological protocol of Benítez et al. [30], a curve with 37 points was defined along the test outline of individuals, beginning and ending at the ambulacrum III (counterclockwise), using the TPSdig2 v. 2.31 [31]. The curve was appended to landmarks using tpsUtil32 [29]. To estimate measurement error, landmark digitization was performed twice at different times. Landmark information was extracted through a Procrustes fit analysis on both measurements combined, which allowed for the standardization of the samples by eliminating the effect of size, position, and rotation [32]. A principal component analysis (PCA) was performed using the shape covariance matrix of individuals to simulate the morphospace. To test for a significant effect of the localities (group) and sex of individuals in shape and size, a Procrustes ANOVA was realized using the Procrustes distances of the covariance matrix. Finally, to evaluate the shape differences, pairwise differences in shape between groups were computed using a permutation test with 10,000 rounds based on the Mahalanobis and Procrustes distances.

In order to evaluate the effect of allometry on shape variation among individuals, a multivariate regression was performed with shape as the dependent variable and the centroid size as the independent variable. The significance of allometry was computed using a permutation statistical test with 10,000 rounds [33]. All statistical analyses were realized using the software MorphoJ v 1.07a [34] and the package “geomorph” [35] of the R version 4.04 software.

## 3. Results

**Figure 2:**
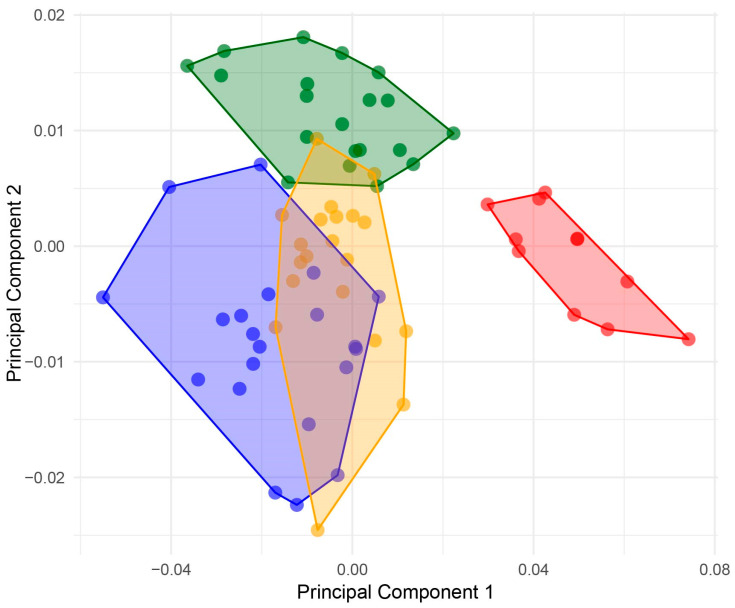
Scatterplot of the first two PCs of the principal component analysis (PCA) of shape variation between *Abatus* groups: *Abatus cordatus* (green), *Abatus cavernosus* from Patagonia (orange), *Abatus agassizii* (blue), and *Abatus cavernosus* from Falklands/Malvinas (red).

**Figure 3:**
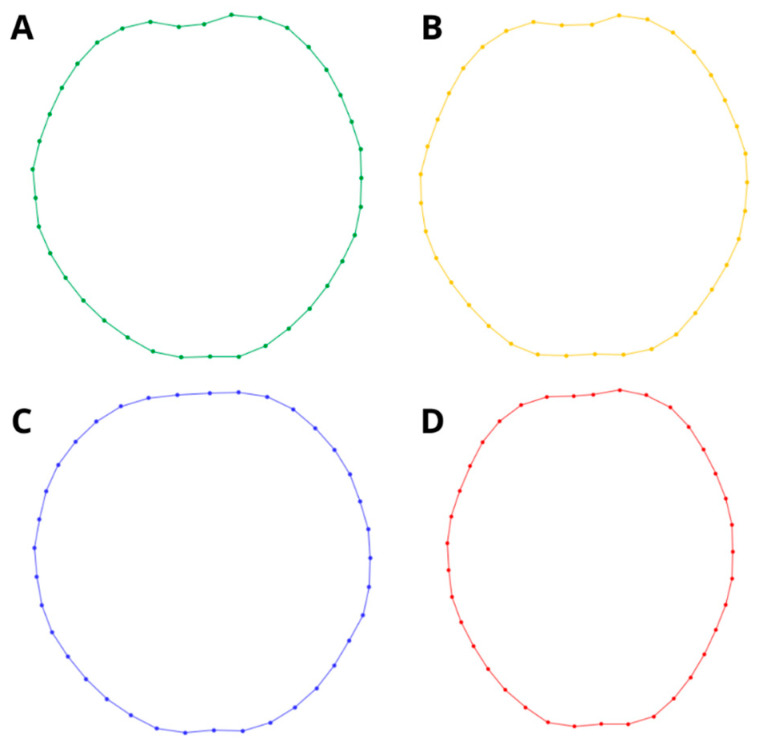
Wireframe representation of groups’ average shape with the respective positioning of landmarks along the test outline, in aboral view: (**A**) *Abatus cordatus* (green), (**B**) *Abatus cavernosus* from Patagonia (orange), (**C**) *Abatus agassizii* (blue), and (**D**) *Abatus cavernosus* from Falklands/Malvinas (red).

The PCA of shape variation between samples showed that 79.19% of the variance is explained by the first two components (PC1 = 68.57%; PC2 = 10.61%). The results showed a clear difference between *A. agassizii*, *A. cordatus*, and *A. cavernosus* from the Falklands/Malvinas, while the shape variation of *A. cavernosus* from Patagonia overlaps with *A. agassizii* and *A. cordatus* and is clearly separated to the *A. cavernosus* from Falklands/Malvinas (Figure 2). The shape varies in relative test length and width along the first PC; meanwhile, along the second axis, shape varies in the anterior part of the test, with individuals having a more or less pronounced anterior sulcus, either reinforcing or reducing the heart-shaped outline of specimens (Figure 3). The Procrustes ANOVA tested a significant effect of locality on test shape and size variation but showed no significant effect of sex (Table 1). Finally, the permutation test of groups evidenced significant pairwise differences in shape (Table 2 and Table 3), with *A. cordatus* and *A. cavernosus* (Patagonia) being the most similar, and *A. agassizii* and *A. cavernosus* (Falklands/Malvinas) the most different.

**Figure 4:**
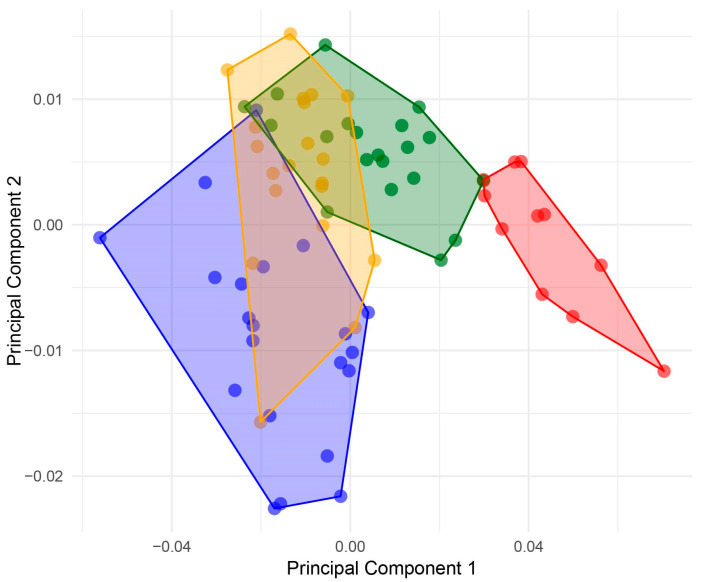
Scatterplot of principal component analysis (PCA) for *Abatus* groups, which shapes the use of the residuals of the multivariate regression (corrected by size). *Abatus cordatus* (green), *Abatus cavernosus* from Patagonia (orange), *Abatus agassizii* (blue), and *Abatus cavernosus* from Falklands/Malvinas (red).

**Figure 5:**
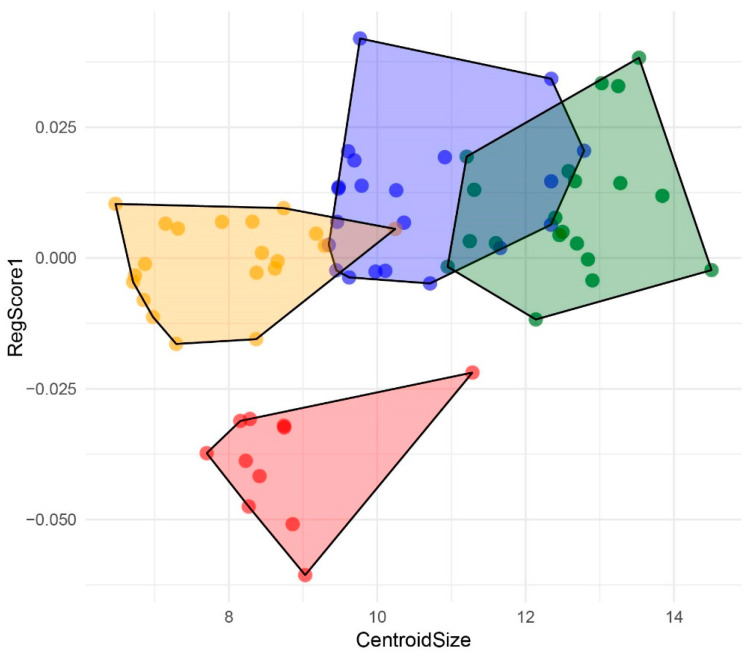
Multivariate regression of the *Abatus* species for the different groups: *Abatus cordatus* (green), *Abatus cavernosus* from Patagonia (orange), *Abatus agassizii* (blue), and *Abatus cavernosus* from Falklands/Malvinas (red).

The influence of centroid size by allometry in four groups of *Abatus* was eliminated from analyses using the multivariate regression residual, correcting PCA analyses by size (Figure 4). In fact, the first and second PCs accumulated 80.11% of the variance (PC1 = 68.83%; PC2 = 11.27%). In addition, the effect of allometry was tested significantly in the four groups (9.8%; *p*-value: <0.0001) that showed a significant relationship between the shape and centroid size of individuals (Figure 5).

## 4. Discussion

The present study showed that geometric morphometrics is a powerful tool to evaluate shape variation in groups and species of *Abatus* from Antarctic and subantarctic provinces. The approach evidenced a clear distinction between species in test outlines supporting the current taxonomy based on the presence, or absence, of a frontal sulcus and on other morphological characters related to globiferous pedicellariae [1]. Interestingly, the results showed no effect of sex in outline shape. Although sexual dimorphism is not a very recurrent feature in echinoids, irregular species show noticeable differences, specifically in the depression of the marsupia (for brood their young) and in the gonopore size [4,16,17]. However, although these traits change the morphology of the female testa, they do not seem to generate an intersexual difference in the outline shape.

The computed morphospace revealed two morphological groups. The first one is composed of the three studied species, where *A. agassizii* and *A. cordatus* differentiate from each other in the development of the anterior sulcus along PC2. *Abatus cavernosus* from Patagonia is intermediate in shape between the two species. *Abatus agassizii* exhibits a more rounded test outline in aboral view, without the heart-shaped form that characterizes other species. In contrast, *A. cordatus* and *A. cavernosus* showed the presence of a deep frontal sulcus, in line with diagnostic characters used for the taxonomy of the three species [1].

Interestingly, a second morphological group corresponding to individuals from the Falklands/Malvinas (morphologically identified as *A. cavernosus*) present a shape markedly different from *A. cavernosus* from Patagonia, *A. agassizii,* and *A. cordatus*. The Falklands/Malvinas group is represented along PC1 by specimens with more elongated and narrower tests, which contrasts with the more rounded shape of the group. This clear difference in shape between *A. cavernosus* from the Falklands/Malvinas and the one from Patagonia can be explained by several factors [6,12]. The difference in shape could be attributed to phenotypic plasticity and peculiar local environmental conditions prevailing in the sampling areas. In burrowing marine invertebrates, factors such as the type and grain size of sediment can affect body shape [36]. Phenotypic plasticity had already been documented in *A. cordatus* and was associated with local environmental conditions [37]. In irregular sea urchins, Ferber and Lawrence [38] found that sediment grain size affects the burrowing capacity of individuals. Similarly, Saitoh and Kanazawa [39] evidenced morphological adaptations of spantagoid body shape and type of appendages, allowing for a more efficient streamline and stability in bottom currents flowing over the sediment surface. Therefore, different local environmental conditions between localities could cause an alteration in development, changing the adult phenotype of individuals [40]. By contrast, there may be other environmental factors such as stress, pollution, or ecological interactions with other species, which may cause instability in development, that is, a variation in the shape of individuals due to random fluctuations during ontogeny [24]. This could be evaluated in the future through an analysis of fluctuating asymmetry [30]. A second hypothesis is that the Falklands/Malvinas group could constitute a differentiated evolutionary unit (different evolutionary trajectory). It is commonly claimed that the Falklands/Malvinas share faunal assemblages with Patagonia. However, genetics and biogeographic studies have detected strong population differences, low gene flow, or even isolation between Patagonia and Falklands/Malvinas in several marine invertebrates with pelagic larvae, e.g., [41,42]. In *Abatus*, a direct developer without a pelagic larval phase, the low mobility of adults and the absence of a dispersal stage considerably limit gene flow between populations even between nearby patches [43]. Therefore, for such shallow coastal sea urchins, the distance between Falklands/Malvinas and eastern Patagonia may have promoted geographic isolation and genetic divergence, leading to the formation of two different genetic and evolutionary units such as previously reported in the brooding isopod *Serolis paradoxa* [44]. Further genetic studies should complement the GM approach and help clarify the identity of this group.

After being released to the sea floor, following the brooding period, juveniles grow constantly in a non-linear way, rather it appears to be a sigmoid curve [45], maintaining the size after a certain age. Mespoulhé [37] mentioned that juveniles present a different shape compared to adults, i.e., a not conservation of shape during development, which is consistent with our results. This evidence opens questions about the role of shape differences through development that could bring adaptive or survival advantages to younger individuals, for example, in locomotion, metabolic rate, or use of habitats [46,47], or due to architectural constraints of development [37].

## 5. Conclusions

This study showed that GM can be powerful in differentiating between the shapes of closely related *Abatus* species. The genomics approach could also help elucidate the intricate relationships between *Abatus* species. In the future, the combination of genomics and morphometrics approaches, together with ecological components, could be used to better understand the diversification and evolutionary relationships among species of *Abatus* across the Southern Ocean.

## Figures and Tables

**Table 1:** Result of the Procustes ANOVA assessing the effect of locality (group) and sex on test shape variation and centroid size. Significant *p*-values are highlighted in bold.

Centroid Size					
**Effect**	**SS**	**MS**	**df**	**F**	*p*
Localities	238.214	79.404681	3	73.83	**<0.0001**
Sex	0.57326	0.57326	1	0.53	0.4679
Individual	72.059	1.075507	67		
**Shape**					
Localities	0.03835	0.0003653	105	46.39	**<0.0001**
Sex	0.00011	0.000003278	35	0.42	0.999
Individual	0.01847	0.000007874	2345	2.77	**<0.0001**

**Table 2:** Pairwise comparisons between the four groups of *Abatus*. Results are reported as Mahalanobis distance after 10,000 permutation rounds. Significant distances are shown by an asterisk.

	*A. agassizii*	*A. cordatus*	*A. cavernosus* (F/M)
*A. cordatus*	12.1293 *		
*A. cavernosus* (F/M)	13.4967 *	9.5791 *	
*A. cavernosus* (P)	10.5548 *	7.9832 *	10.1073 *

**Table 3:** Pairwise comparison between the four groups of *Abatus*. Results are reported as Procrustes distances after 10,000 permutation rounds. Significant distances are shown by an asterisk.

	*A. agassizii*	*A. cordatus*	*A. cavernosus* (F/M)
*A. cordatus*	0.0244 *		
*A. cavernosus* (F/M)	0.0657 *	0.0537 *	
*A. cavernosus* (P)	0.0191 *	0.0171 *	0.0527 *

## Data Availability

Data will be made available by personal request to the corresponding author.
